# Pharmacokinetics and bioavailability of single dose ibuprofen and pseudoephedrine alone or in combination: a randomized three-period, cross-over trial in healthy Indian volunteers

**DOI:** 10.3389/fphar.2014.00098

**Published:** 2014-05-09

**Authors:** Prashant Kale

**Affiliations:** Lambda Therapeutic Research Ltd.Ahmedabad, India

**Keywords:** bioequivalence, ibuprofen, NSAIDs, pseudoephedrine, pharmacokinetics

## Abstract

**Objective:** To compare the bioavailability of single dose ibuprofen 200 mg and pseudoephedrine hydrochloride 30 mg administered alone or in combination as an oral suspension.

**Methods:** This was a single-center, randomized, single-dose, open-label, 3-period, crossover study. After an overnight fast (≥10 h), 18 healthy male subjects received either ibuprofen 200 mg (reference-A), pseudoephedrine 30 mg (reference-B) or the combination (test-C) as a suspension, on 3 separate visits, with blood sampling up to 36-h post-dose. The primary pharmacokinetic parameters, maximum plasma concentration (C_max_) and area under the plasma concentration–time curve (AUC) from time zero to last measurable concentration (AUC_0−t_) and extrapolated to infinity (AUC_0−∞_) were compared by an analysis of variance using log-transformed data. Bioequivalence was concluded if the 90% confidence intervals (CIs) of the adjusted geometric mean (gMean) ratios for C_max_ and AUC were within the predetermined range of 80–125%, in accordance with regulatory requirements.

**Results:** For the test formulation, the ibuprofen gMean C_max_ was 17.0 μg/mL (vs. 18.1 μg/mL for reference-A), AUC_0−t_ was 57.1 (vs. 60.0 μg·h/mL), and AUC_0−∞_ was 59.9 μg·h/mL (vs. 63.1 μg·h/mL). The 90% CIs for the ratio (test/reference-A) were 81.0–108.1% for C_max_, 91.5–98.4% for AUC_0−t_ and 91.6–97.9% for AUC_0−∞_. For pseudoephedrine, the gMean C_max_ for the test formulation was 97.2 ng/mL (vs. 98.5 ng/mL for reference-B), AUC_0−t_ was 878.4 (vs. 842.8 ng·h/mL) and AUC_0−∞_ was 907.8 ng·h/mL (vs. 868.3 ng·h/mL). The 90% CIs for the ratio (test/reference-B) were 92.4–106.9% for C_max_, 97.7–111.0% for AUC_0−t_ and 97.9–111.3% for AUC_0−∞_. All treatments were well tolerated.

**Conclusion:** This oral suspension containing ibuprofen and pseudoephedrine combined in a new formulation met the regulatory criterion for bioequivalence compared with oral suspensions containing the individual components.

## Introduction

Ibuprofen and pseudoephedrine are commonly used for symptomatic management of the common cold, sinusitis and influenza. The benefit from these agents is due to a combination of analgesic, antipyretic and anti-inflammatory effects of ibuprofen and decongestant effects of pseudoephedrine (Anonymous, 1999b; Rainsford, 2013). Both agents are rapidly absorbed after oral administration, with peak plasma concentrations (C_max_) achieved within 1–2 h for ibuprofen and after 1–3 h for pseudoephedrine (Davies, 1998; Anonymous, 1999a,b). Taken together, it is likely that there would be potential for improved symptomatic relief with a combination product, subject to demonstration of bioequivalence. Various formulations of combined ibuprofen and pseudoephedrine, including oral tablets, slow-release capsules and liqui-gel suspensions have been studied and approved for clinical use in adults and children. The aim of this study was to compare the pharmacokinetics of an oral suspension containing ibuprofen and pseudoephedrine combined in a new formulation vs. oral suspensions containing the individual components at the same doses.

## Subjects and methods

This was a single-center (Lambda Therapeutic Research Ltd, Ahmedabad, India), randomized, single-dose, open-label, 3-treatment, 3-period, 6-sequence, crossover trial conducted in healthy volunteers between June and July 2003. The study protocol was approved by an independent ethics committee, and was conducted in accordance with local regulations and the Declaration of Helsinki and Good Clinical Practice. Written informed consent was obtained from all subjects before performing any trial-related activities. The study design was in accordance with the European Medicine Agency note for guidance on the investigation of bioavailability and bioequivalence of fixed combination products (European Medicines Agency, 2002) and US Food and Drug Administration guidance for industry on the bioavailability and bioequivalence of oral drugs (US Food and Drug Administration, 2003).

### Subjects

Healthy male subjects aged 18–55 years with a body mass index (BMI) of 18–25 kg/m^2^ were considered for the study. Subjects had no significant disease (gastrointestinal, hepatic, renal, respiratory, cardiovascular, metabolic, skin, immunological, or hormonal), clinically significant medical history, or abnormal ECG, vital signs or laboratory values at screening 21 days before dosing. Additional exclusion criteria included allergy to ibuprofen, pseudoephedrine, or other non-steroidal anti-inflammatory drugs (NSAIDs), history of asthma, nasal polyp or NSAID-induced urticaria or drug or alcohol abuse; subjects who smoked >10 cigarettes per day or could not abstain during the study, who participated in another trial within 90 days, or used any other medication within 14 days of enrolment were also excluded. Subjects were tested for drug and alcohol consumption prior to each study period.

### Study design and treatments

**Figure 1:**
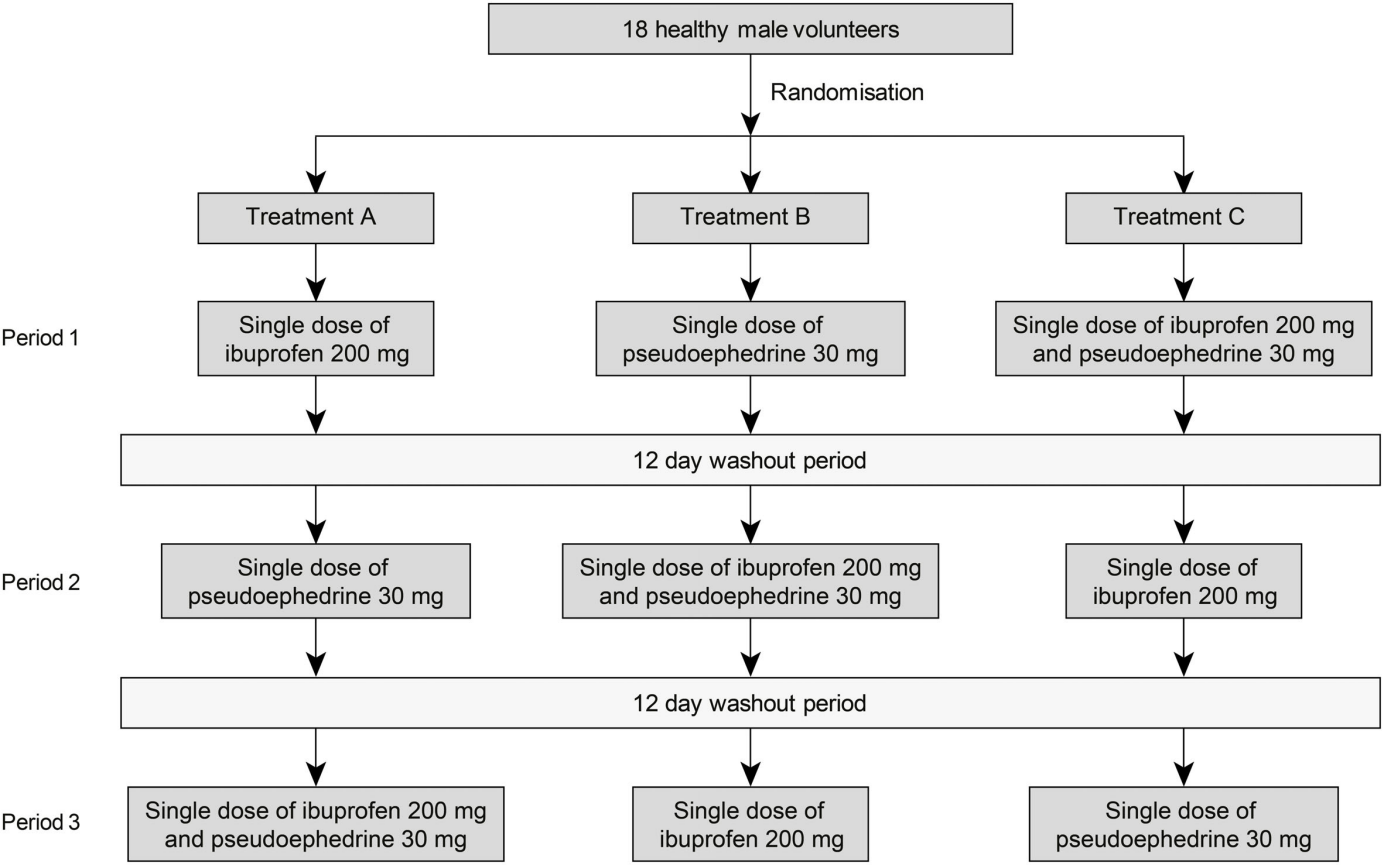
**Study design**.

The study comprised a screening period followed by 3 treatment periods, each separated by a 12-day washout period. Subjects were randomly assigned using a computer-generated list to receive one of 6 possible treatment sequences for single dose ibuprofen (200 mg suspension, 100 mg/5 mL, reference A), single dose pseudoephedrine hydrochloride (30 mg suspension, 15 mg/5 mL, reference B) or the combination of ibuprofen 200 mg and pseudoephedrine 30 mg (suspension, test C) (Figure 1). All treatments were manufactured by Orbis Consumer Products Limited, Middlesex, United Kingdom.

Subjects attended the study center on the evening before dosing. After fasting overnight (≥10 h), subjects were dosed orally (10 mL suspension with 240 mL of water) in the sitting position by study personnel using a syringe. The syringe was then rinsed with water and administered again with any remaining water. Administration was confirmed by a mouth-check. Water was allowed *ad libitum* except 1 h before and 2 h after dosing. Subjects were seated or in ambulatory posture for 3 h post-dose and fasted for 4 h after dosing. During each period subjects remained in the study center until blood samples had been taken 20 h (ibuprofen alone) and 36 h (pseudoephedrine alone and ibuprofen-pseudoephedrine) after dosing. Xanthine-containing food or beverages, alcohol, extreme physical activity, cigarettes and tobacco products were not permitted 48 h before and during post-dose blood sampling.

### Pharmacokinetic evaluation

Venous blood samples (6 mL for each reference product, 8 mL for the test product) for measurement of plasma concentrations of ibuprofen and pseudoephedrine were collected in heparinized vacutainers via an indwelling cannula during each study period before and at 10, 20, 40, 50, and 60 min of the first hour, and thereafter every 20 min for up to 3 h, and 3.5, 4, 6, 8, 10, and 12 h after dosing. Additional samples were collected at 16, 24, 30, and 36 h after dosing with treatments B and C. Samples were centrifuged for 15 min at 10°C (2960 rpm) and plasma aliquots were taken and frozen (−20°C then −60°C) until analysis.

Plasma concentrations of ibuprofen and pseudoephedrine were analyzed separately using liquid chromatography mass spectrometry procedures, which were fully validated and developed from methods previously used by Lambda Therapeutic Research Ltd, Ahmedabad, India (Lambda, Data on File). Briefly, ibuprofen plasma concentrations were measured following protein precipitation extraction (internal standard diclofenac) by high pressure liquid chromatography (HPLC) using a Kromasil® C18 250 × 4.6 mm, 5 μm column (mobile phase 28% acetonitrile and 72% buffer pH 6.8). The limit of quantification was 0.730 μg/mL. Pseudoephedrine plasma concentrations were analyzed following liquid-liquid extraction (carbamazepine as the internal standard) by liquid chromatography-mass spectrometry (LC-MS/MS) analysis using a 50 × 4.6 mm Chromolith® SpeedROD RP-18e column (mobile phase 90% methanol and 10% 2 mM ammonium acetate buffer, pH 3.5). The limit of quantification was 2.053 ng/mL. Assay performance was assessed by back-calculation of calibration standards, tabulation of the standard curve fit function parameters and measurement of quality control samples. Validation data documented adequate accuracy, precision and specificity of the liquid chromatography mass spectrometry assays employed for the study.

### Safety evaluation

Clinical examination findings and vital signs (pulse, blood pressure) were reported on admission and before discharge from the study center in all 3 study periods. Vital signs were measured 4, 8, and 12 h (for all treatments) and additionally 24, 30, and 36 h (for treatments B and C) after dosing. Subjects were questioned for well-being at the time of clinical assessments. Adverse events were collected during each study period with severity (mild, moderate or severe) and investigator assessment of the relationship to the study medication (definite, possible, doubtful, or none).

### Pharmacokinetic analyses

The primary variables were the area under the plasma concentration-time curve from time 0 to the last quantifiable data point (AUC_0−t_), from time zero extrapolated to infinity (AUC_0−∞_) and C_max_. Time of maximum exposure (t_max_) was a secondary variable. Non-compartmental analysis of plasma concentration-time data was performed using WinNonlin® Professional software (Version 4.0.1, Pharsight Corporation, Cary, NC, USA). All values below the limit of quantification were considered as zero for pharmacokinetic analysis.

### Statistical analyses

Subjects were simultaneously enrolled in the study so as to ensure that at least 18 subjects were dosed at the beginning of the study. This number of subjects was considered appropriate for this type of study. In case of drop-outs, samples from subjects who completed at least 2 periods of the study were analyzed, provided they had completed treatment with the test product.

Statistical analyses were performed using PROC MIXED (SAS®, version 8.2, SAS Institute Inc., Cary, NC, USA). AUC_0−∞_, AUC_0−t_, and C_max_ data for ibuprofen and pseudoephedrine were log-transformed and compared between treatment groups using an analysis of variance (ANOVA) model with sequence, subjects within sequences, period and treatment as sources of variation. The least square means and 90% confidence intervals (CI) were calculated and then back-transformed to the original scale to provide the point estimator and interval estimates for the geometric mean (gMean) of intra-subject test/reference ratio. In accordance with regulatory requirements (European Medicines Agency, 2002; US Food and Drug Administration, 2003), bioequivalence was concluded if the 90% CIs were within the range 80–125%. For all other parameters, descriptive statistics were presented. T_max_ was compared using the Wilcoxon signed rank test. Intra-subject variability and power to detect a 20% mean difference between formulations were calculated for log-transformed pharmacokinetic parameters using root mean square error computed by PROC MIXED.

## Results

### Participants

Nineteen healthy Indian male subjects were enrolled and 18 subjects aged 18–39 years were randomized; one subject withdrew before dosing and was subsequently replaced. The participant demographic characteristics are summarized in Table 1. Fifteen subjects completed all 3 dosing periods. Three subjects discontinued the study due to an adverse event, protocol violation, or withdrawal for personal reasons; 2 of these subjects received both pseudoephedrine (reference B) and the combination (test C) before withdrawal and were therefore included in pharmacokinetic analyses for these treatments.

**Table 1:** **Baseline characteristics of the randomized male study population (*n* = 18)**.

**Characteristic**	
Age [years]	25.9 ± 6.2 (18-39)
Weight [kg]	57.8 ± 5.2
BMI [kg/m^2^]	20.6 ± 1.9

*Data shown as mean ± standard deviation (range)*.

### Pharmacokinetic profiles

**Figure 2:**
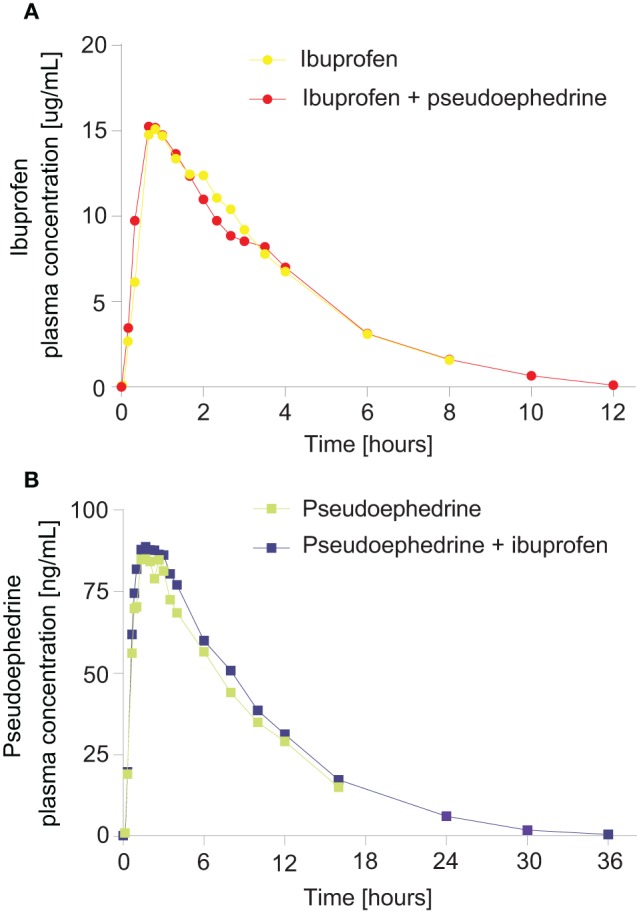
**Geometric mean plasma concentration vs. time curves for (A) ibuprofen from an ibuprofen/pseudoephedrine combination vs. ibuprofen alone, (B) pseudoephedrine from a pseudoephedrine/ibuprofen combination vs. pseudoephedrine alone**.

The ibuprofen and pseudoephedrine plasma concentration-time profiles are shown in Figure 2 and pharmacokinetic parameters are summarized in Tables 2, 3.

**Table 2:** **Pharmacokinetic parameters of ibuprofen 200 mg, either alone (reference A), or in combination with pseudoephedrine 30 mg (test, C) (*n* = 15)**.

**Parameter and unit**	**Ibuprofen (*n* = 15)^a^**		**Adjusted gMean ratios (90% CI) (%)^c^**	**Intra subject gCV [%]^c^**
	**Alone (Reference, A)**	**+pseudoephedrine (Test, C)**		
AUC_0−t_[μg·h/mL]	60.0 (14.3)	57.1 (19.0)	94.9 (91.5–98.4)	5.6
AUC_0−∞_ [μg·h/mL]	63.1 (14.6)	59.9 (18.1)	94.7 (91.6–97.9)	5.1
C_max_ [μg/mL]	18.1 (21.9)	17.0 (27.9)	93.6 (81.0–108.1)	22.3
t_max_ [h]^b^	0.83 (0.67–2.67)	0.83 (0.67–3.50)		
t_½_[h]	1.96 (15.4)	2.00 (14.6)		

a *Results are presented as geometric mean (CV%) unless stated*.

b *These values are expressed as median (range)*.

c *Log-transformed data, ratio of least-square geometric means (test/reference) based on ANOVA model*.

*AUC_0−∞_ area under the concentration-time curve from zero time to infinity, AUC_0−t_ area under the plasma concentration-time curve from zero time to time of the last quantifiable drug plasma concentration, C_max_ maximum observed plasma concentration, CI confidence interval, CV coefficient of variation (%), gMean geometric mean, t_max_ time to reach C_max_, t_½_ terminal half-life*.

**Table 3:** **Pharmacokinetic parameters of pseudoephedrine 30 mg either alone (Reference A) or in combination with ibuprofen 200 mg (Test, C) (*n* = 17)**.

**Parameter and unit**	**Pseudoephedrine (*n* = 17)^a^**		**Adjusted gMean ratios (90% CI) (%)^c^**	**Intra subject gCV [%]^c^**
	**Alone (Reference, B)**	**+ibuprofen (Test, C)**		
AUC_0−t_ [ng·h/mL]	842.8 (34.5)	878.4 (267)	104.1 (97.7–111.0)	10.6
AUC_0−∞_ [ng·h/mL]	868.3 (34.2)	907.8 (25.9)	104.4 (97.9–111.3)	10.6
C_max_ [ng/mL]	98.5 (21.1)	97.2 (21.4)	99.4 (92.4–106.9)	12.0
t_max_ [h]^b^	1.67 (0.67–3.0)	2.00 (0.83–3.0)		
t_½_ [h]	4.7 (18.4)	4.9 (16.0)		

a *Results are presented as geometric mean (CV%) unless stated*.

b *These values are expressed as median (range)*.

c *Log-transformed data, ratio of least-square geometric means (test/reference) based on ANOVA model*.

*AUC_0−∞_ area under the concentration-time curve from zero time to infinity, AUC_0−t_ area under the plasma concentration-time curve from zero time to time of the last quantifiable drug plasma concentration, C_max_ maximum observed plasma concentration, CI confidence interval, CV coefficient of variation (%), gMean geometric mean, t_max_ time to reach C_max_, t_½_ terminal half-life*.

#### Ibuprofen

Ibuprofen was rapidly absorbed, with a median t_max_ of 50 min (either alone or in combination, *p* = 0.67) (Table 2). The plasma concentration-time curves of ibuprofen (with or without pseudoephedrine) showed a parallel decline in distribution and elimination phases (Figure 2A). The gMean values of C_max_, AUC_0−t_ and AUC_0−∞_ for ibuprofen were similar when given alone or in combination with low interindividual variation (CV ranged from 14.3 to 27.9%). Bioequivalence was demonstrated as the 90% CIs of the ratios of point estimates (test/reference) for C_max_, AUC_0−t_, and AUC_0−∞_ were within the range of 80–125%.

#### Pseudoephedrine

Pseudoephedrine was rapidly absorbed, with a median t_max_ of 100 and 120 min for the combination and when given alone, respectively (*p* = 0.92) (Table 3). The plasma concentration-time curves of pseudoephedrine (with or without ibuprofen) showed a similar parallel decline in distribution and elimination phases (Figure 2B). The gMean values of C_max_, AUC_0−t_, and AUC_0−∞_ for pseudoephedrine were similar when given alone or in combination with low interindividual variation (CV ranged from 21.1 to 34.5%). Bioequivalence was demonstrated as the 90% CIs of the point estimates (test/reference) for C_max_, AUC_0−t_, and AUC_0−∞_) were in the range 80–125%.

### Safety results

A total of 14 treatment-emergent adverse events were reported by 9 subjects (3 for the test formulation, 6 for ibuprofen alone and 5 for pseudoephedrine alone). All adverse events were mild and resolved spontaneously. Two events were considered as possibly related to the test medication (test C). One subject reported forearm itching which was considered definitely related; the event was also reported with pseudoephedrine alone and was also considered possibly related to the study drug. Another subject reported heartburn which was considered as possibly related to the study medication. In addition, itching and generalized body ache were reported as possibly related to reference A (ibuprofen alone). There were no clinically relevant changes in vital signs, ECGs or laboratory findings.

## Discussion

The present study confirmed that the rate (C_max_ and t_max_) and extent (AUC) of absorption of ibuprofen or pseudoephedrine from a suspension formulation were similar when administered as single doses of the individual components and when administered in combination in healthy volunteers. The 90% CIs for the treatment ratios (combination/individual treatments) for C_max_, AUC_0−t_, and AUC_0−∞_ of ibuprofen and pseudoephedrine were within the range of 80–125% and therefore satisfied regulatory criteria for bioequivalence (European Medicines Agency, 2002; US Food and Drug Administration, 2003).

For both oral ibuprofen 200 mg and pseudoephedrine hydrochloride 30 mg, AUC_0−t_ and C_max_ in this study (see Tables 1, 2) were similar to those reported for a film-coated tablet (58.6 μg·h/mL and 14.8 μg/mL, respectively for ibuprofen; 1080 ng·h/mL and 139 ng/mL for pseudoephedrine) (Boehringer Ingelheim, 2011). Further studies show that multiple dose administration of combined ibuprofen and pseudoephedrine, either as modified-release capsules in healthy adult males or as an oral suspension in children, has a similar pharmacokinetic profile as compared with single-ingredient formulations (Stillings et al., 2003; Gelotte et al., 2010).

Single doses of ibuprofen and pseudoephedrine were well-tolerated when given alone and in the combination treatment to healthy male volunteers. Itching reported for one patient treated with pseudoephedrine alone or in combination with ibuprofen was consistent with the established tolerability profile of pseudoephedrine (Anonymous, 1999b).

In summary, this ibuprofen-pseudoephedrine combination formulation met the regulatory criterion for pharmacokinetic bioequivalence compared with concurrent administration of the individual components.

### Conflict of interest statement

This study was sponsored by Orbis Consumer Products Limited, United Kingdom. The author is an employee of Lambda Therapeutic Research, which was contracted by Orbis Consumer Products as the Clinical Research Organisation for the conduct of this study and received financial support for its services. The author meets the criteria for authorship as recommended by the International Committee of Medical Journal Editors (ICMJE) and was fully responsible for the analysis and interpretation of the data.
